# Neuropeptide Receptors: Novel Targets for HIV/AIDS Therapeutics

**DOI:** 10.3390/ph4030485

**Published:** 2011-03-09

**Authors:** Donald R. Branch

**Affiliations:** 1 Department of Medicine, University of Toronto, Toronto, Ontario M5G 2M1, Canada; E-Mail: don.branch@utoronto.ca; Tel.: +1-416-313-4458; Fax: +1-416-974-9757; 2 Department Laboratory Medicine and Pathobiology, University of Toronto, Toronto, Ontario M5G 2M1, Canada; 3 Research & Development, Toronto Immunology Hub, Canadian Blood Services, Toronto, Ontario M5G 2M1, Canada

**Keywords:** neuropeptide receptors, HIV/AIDS, VPAC1, VPAC2, HIV therapeutics

## Abstract

The vasoactive intestinal peptide/pituitary adenylyl cyclase-activating polypepetide (VPAC) receptors are important for many physiologic functions, including glucose homeostasis, neuroprotection, memory, gut function, modulation of the immune system and circadian function. In addition, VPAC receptors have been shown to function *in vitro* to modulate the infection of HIV by a signal transduction pathway that appears to regulate viral integration. In this article, the affects of VPAC stimulation on HIV infection will be reviewed and approaches for the development of HIV/AIDS therapeutics that target these receptors will be described. Novel HIV/AIDS therapeutics are urgently required to stem the continued spread of this disease, particularly in underdeveloped countries. Drug design to inhibit signaling through VPAC1 and stimulate signaling through VPAC2 could lead to alternative therapies for the treatment and/or prevention of HIV/AIDS.

## 1. Introduction

It has been 30 years since the first published description of acquired immune deficiency syndrome (AIDS) [1]. Unfortunately, there remains no cure for AIDS. However, over this long period of time, much has been learned, and continues to be learned, about the retrovirus causing AIDS, human immunodeficiency virus (HIV). This RNA-containing virus requires virally-derived proteins that include reverse transcriptase, integrase and protease, to successfully complete its lifecycle. This knowledge has led scientists to develop highly active antiretroviral therapy (HAART) which has been exceedingly successful in prolonging life of HIV-infected individuals. However, HAART is expensive and, thus, unavailable to the majority of those infected with HIV, which remains endemic in Sub-Saharan Africa and is increasing in South Asia and Asian countries [2]. Thus, HIV infection and AIDS cases continue to increase worldwide. According to the World Health Organization, as many as 9,000 new cases of HIV infection occur daily and it is projected that by the end of the next decade there will be some 100 million infected individuals [2]. Thus, despite 30 years of research and other efforts to control its spread [3,4], HIV/AIDS remains an ever increasing threat to global health. Development of a suitable vaccine continues to be years in the future. Therefore, in order to avert increased morbidity and mortality, we need to develop new HIV/AIDS therapeutics and we need to do this with urgency.

## 2. Vasoactive Intestinal Peptide/Pituitary Adenylyl Cyclase-Activating Polypeptide (VPAC) Receptors

Several receptors that can interact with structurally related regulatory neuroendocrine peptides that include vasoactive intestinal peptide (VIP), secretin, pituitary adenylyl cyclase-activating polypeptide (PACAP), glucagon, gastric inhibitory peptide (GIP) and growth hormone releasing factor (GRF) have been described [5-7]. These neuroendocrine receptors belong to the group B sub-family of seven transmembrane G protein-coupled receptors referred to as the secretin family of neuroendocrine receptors [5-7]. There are three members of this receptor family [5]: VPAC1, VPAC2, and PAC1. VPAC1 is widely expressed on cells and tissues, present in liver, intestine, kidney, and in certain regions of the brain, notably the cortex, hippocampus, and olfactory bulb, as well as on immune T cells and monocytes. VPAC2 is not as widely expressed as VPAC1, found mostly in the central nervous system, in the thalamus, hippocampus, suprachiasmatic nucleus and hypothalamus. However, VPAC2 is expressed on epithelial cells and is inducible on T lymphocytes and monocytes. Indeed, VPAC receptors have been shown to be differentially expressed in monocytes and T lymphocytes. VPAC1 is expressed at higher levels in resting compared to activated CD4^+^ T lymphocytes and is expressed in monocytes. VPAC2 is expressed optimally on activated CD4^+^ T lymphocytes and monocytes [8-10].

The two VPAC receptors, although structurally-related, exhibit distinct structure-function differences [10-12]. Indeed, secretin, a VIP/PACAP-related protein, has significant biological activity on VPAC1 but is relatively inactive on VPAC2 [13]. In contrast, the lizard venom-derived protein, helodermin, from the Gila monster [14], has significant biological activity on VPAC2 and no or little activity on VPAC1 [15,16]. Moreover, though VIP binds to both receptors with similar affinity and both VPAC1 and VPAC2 generated signals have been shown to result in increased [cAMP]i and [Ca ^2+^]i [7], signal transduction through these receptors can result in different functional outcomes [8,12]. In this regard, VPAC receptors may be similar to angiotensin II type 1 and type 2 receptors [17,18]. This has led to speculations and some supportive evidence that these receptors generate additional and different signaling cascades [7,10] but, also, that the differential expression of these two receptors on cells that express both receptors may result in cross talk that may influence the outcome of the signal transduced [12].

Investigators have previously alluded to a possible role for VIP in HIV infection [19-24]. VIP, produced during immune activation and at particularly high levels in the gastrointestinal tract, has been shown *in vitro* to directly activate transcription from the HIV-1 long terminal repeat (LTR) promoter regions [20]. Early reports claimed antibodies were present in HIV infected individuals that showed spectral and sequence homologies to VIP and peptides derived from HIV gp120 that bound these antibodies [21]. Also, antibodies to a VIP-like structure within HIV gp120 were found in HIV infected individuals that did not progress to AIDS [22]. Our laboratory has shown that anti-VIP can immunoprecipitate HIV [23], suggesting that HIV gp120 may directly interact with the receptor for VIP on the cell surface of T-cells and monocytes, targets for HIV infection. Thus, if HIV can mimic VIP, it may allow HIV to bind to the receptor for VIP on resting T cells or monocytes; this may provide some advantage to the virus. Indeed, our laboratory has shown that antibodies that specifically block the signal transduced by VPAC1 but not ligand binding, can inhibit HIV infection by as much as 80% [23]. Thus, a portion of HIV gp120 appears to resemble VIP and, through this mimicry, can interact and activate the VPAC1 receptor, providing a facilitation effect for HIV infection.

Our recent results show VPAC1 signaling facilitates the integration of the viral cDNA of HIV, possibly through activation of a tyrosine kinase [10,24] that may be responsible for tyrosine phosphorylation of the HIV matrix protein allowing formation of the pre-integration complex [25-27].

## 3. The Role of VPAC1 in HIV Infection

Our laboratory and others have shown that VIP can activate transcription of the HIV LTR promoter [20]. However, we are the only group that has examined a role for VPAC receptors in HIV infection. We have shown VPAC1 to play a significant role in facilitating the HIV infection [23,24]. Importantly, we have shown that blocking of the VPAC1 signaling pathway results in significant inhibition of HIV infection [23]. We have demonstrated that HIV can facilitate its own infection by interacting with and stimulating VPAC1 [23,25]. We confirmed the significant role played by VPAC1 in HIV infection by over-expressing VPAC1 in HIV susceptible target cells and by using antisense to knock-down VPAC1 expression [23]. Initial studies suggested that the mechanism of VPAC1 signaling is to facilitate viral integration [23] and recent preliminary studies support this hypothesis [25]. These studies combined indicate that HIV does not use VPAC1 to gain entry into the host cells but, instead, uses VPAC1 signaling for inducing the integration of the viral cDNA. Thus, stimulation of VPAC1 either by specific ligand or by HIV itself provides an activation signal that is not required for viral entry but may be necessary for successful viral integration into the host genome.

**Figure 1:**
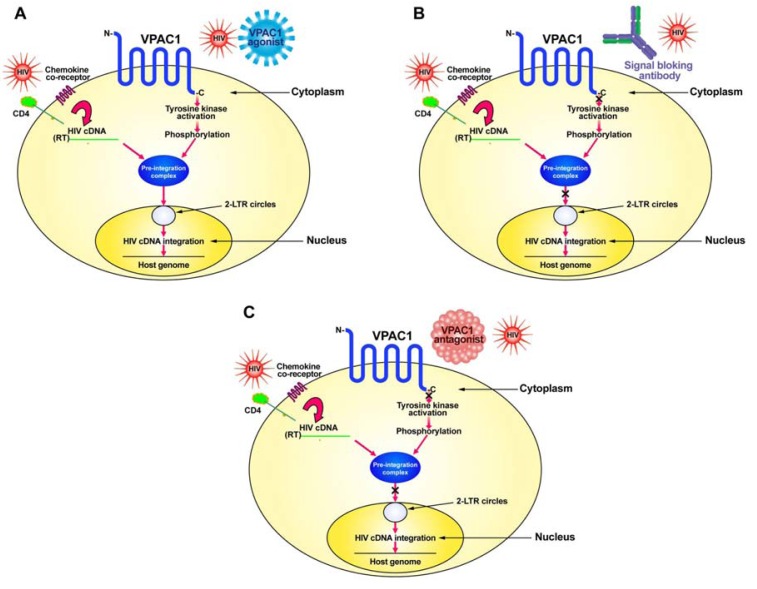
Schematic representation of how HIV infects cells and how VPAC1 therapeutics might work to prevent productive HIV infection. (**A**). Schematic of HIV infection and role of VPAC1; (**B**). Blocking antibody to VPAC1 inhibits the signal transduction and activation of a tyrosine kinase; thus, preventing formation of the pre-integration complex, nuclear transport and integration of the viral cDNA; (**C**). Specific VPAC1 antagonists, either a peptide or small-molecular-weight non-peptide inhibitor, prevent activation of the VPAC1 receptor resulting in inhibition of viral cDNA transport and integration.

HIV normally infects CD4^+^ T-cells through the interaction of its envelope protein, gp120, with cell-surface expressed CD4 and a chemokine co-receptor, either CXCR4 or CCR5 (Figure 1). This interaction allows for HIV to fuse to the cell membrane where it then can deposit its RNA and viral proteins into the cytoplasm of the cell. Using its reverse transcriptase (RT), the viral RNA is converted into its complementary DNA (cDNA). The viral cDNA then forms a pre-integration complex with the viral proteins, p17 (matrix protein), integrase and Vpr. In order to form the pre-integration complex, the matrix protein must be phosphorylated on tyrosine [26,27]. We hypothesize this to be the result of a signal sent through the VPAC1 receptor, either by a specific ligand, such as secretin or VIP, or by HIV itself which has amino acid sequence similarity within its gp120 to VIP [10,23,25]. This tyrosine kinase activation through VPAC1 phosphorylates the matrix protein and allows the formation of the pre-integration complex, which then can move into the nucleus where it forms 2-LTR circular DNA (2-LTR circles) as well as integrates its cDNA into the host genome (Figure 1).

Although the exact tyrosine kinase responsible for the phosphorylation of the matrix protein has yet to be elucidated, the VPAC1-stimulated activation of tyrosine kinase activity that we have observed [10] and especially the c-Src tyrosine kinase activated through VPAC1 [24], seems a good candidate for further study.

## 4. The Role of VPAC2 in HIV Infection

To investigate the possible role of VPAC2 in HIV infection, we initially used the peptide helodermin that preferentially stimulates VPAC2 [14,15]. Helodermin is a 38-kilodalton polypeptide that is derived from the venom of a lizard called the Gila monster (*Heloderma suspectum*). Gila monsters are found only in the Southwestern deserts of Utah, Nevada, California, New Mexico, and in Northern Mexico. Although derived from a venomous lizard, helodermin has been used in studies by neuroscience investigators for many years without reports of toxicity. Based on the amino acid sequence of helodermin, synthetic peptide analogues have been produced having increased affinity for VPAC2 over VPAC1 or PAC1 [16,28,29]. Initially, we found that a single treatment of Jurkat T-cells with helodermin prior to HIV-1 infection results in a significant inhibition of productive infection [30]. This effect was shown to be dose-dependent. Using additional, more specific agonists for VPAC2 confirmed our results using helodermin. These agonists known as RO-25-1553 [16], R3P66 or BAY55-9837 [28], and R3P55 [29] all were shown to inhibit productive HIV infection using 10^−9^M daily administration to cell cultures [30]. Subsequent studies showed that stimulation of VPAC2 but not VPAC1 or PAC1 inhibited HIV infection through prevention of HIV viral cDNA integration into the host mammalian DNA [30].

In contrast to VPAC1 (Figure 1), specific stimulation of the VPAC2 receptor by appropriate agonists sends a signal which inhibits the transport of the pre-integration complex into the nucleus, preventing formation of 2-LTR circles and viral cDNA integration into the host genome [25,30]. We have evidence that VPAC2 stimulation activates a protein tyrosine phosphatase [25]. This tyrosine phosphatase may act to dephosphorylate the matrix protein thereby preventing the formation of the pre-integration complex and preventing viral cDNA nuclear transport and integration.

## 5. Differential VPAC Expression on Cells Targeted by HIV

It is known that on immune cells such as CD4^+^ T-cells, the primary target of HIV infection, that VPAC1 and VPAC2 are differentially expressed [8-10]. On resting, naïve CD4^+^ T-cells, VPAC1 is expressed at high levels with little or no VPAC2 expression [9,10]. Thus, during initial HIV infection of resting CD4^+^ T-cells, HIV would have an advantage as it would bind to and stimulate the VPAC1 receptor with little or no interaction with VPAC2; although, it remains unclear whether HIV can bind to both VPAC1 and VPAC2. Preferential HIV stimulation of VPAC1 would allow for the provision of the signal necessary to form the pre-integration complex. After T cell activation, however, the level of VPAC1 expression drops off sharply while the expression of VPAC2 increases several-fold higher than VPAC1 [9,10]. At this stage, ligands that specifically stimulate VPAC2 would be expected to provide signals that would protect against integration of the viral cDNA. Even VIP which can stimulate both VPAC1 and VPAC2 may preferentially stimulate VPAC2 due to its increased expression on activated T-cells; and thus, could provide some protection at this time. However, as no natural human homologue for helodermin has yet been identified [31], inhibition of HIV infection of CD4^+^ T-cells *in vivo* through VPAC2 activation may not occur naturally as a response to HIV infection. Thus, VPAC2 provides a good candidate target for the development of synthetic agonists to be used in the prevention of HIV infection.

## 6. Potential Therapeutics Targeting VPAC1

In order to develop potential therapeutics that target VPAC1 one must inhibit the signaling pathway through this receptor (Figure 1). This can be done in either of two approaches: (1) produce a humanized monoclonal blocking antibody for VPAC1 receptor signaling; or (2) develop specific peptide agonists of VPAC1 or use a drug discovery approach to identify small-molecular-weight non-peptide compounds that can bind to VPAC1 and prevent signaling.

## 7. Potential Therapeutics Targeting VPAC2

**Figure 2:**
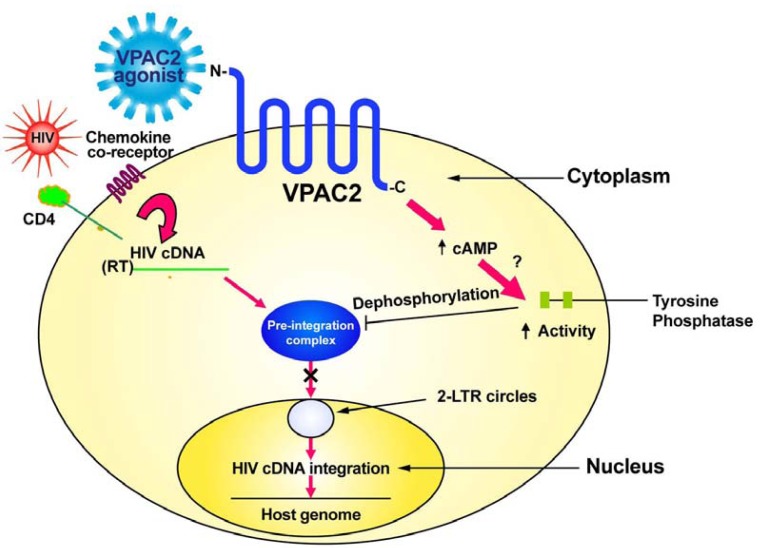
Schematic representation of how VPAC2 therapeutics might work to inhibit HIV infection. In contrast to VPAC1 (Figure 1), specific stimulation of the VPAC2 receptor by appropriate agonists sends a signal which activates a protein tyrosine phosphatase that results in the inhibition of the formation of the pre-integration complex, preventing formation of 2-LTR circles and viral cDNA integration into the host genome [25,30].

Therapeutics to target VPAC2 need to be specific agonists that activate this receptor. Helodermin is a molecule that can activate VPAC2 signaling and inhibit HIV integration. There is no known human homologue of helodermin [31]; however, a number of synthetic peptide analogues having higher affinity than helodermin for VPAC2 compared to VPAC1 have been produced [25,28,29,32] and some of these have shown ability to inhibit HIV infection *in vitro* [30]. Other VPAC2 agonists having even higher specificity and stimulatory ability have more recently been described [33-35]. A pharmacological approach to determine if any of these VPAC2 agonists have *in vivo* toxicities is imperative as it is possible that Phase I/II clinical trials for the use of these compounds for reasons other than HIV/AIDS therapy may soon go forward [33] and show one or more to have low toxicity. This would mean that these compounds could be tested in pre-clinical animal trials for efficacy against HIV infection, including mucosal transmission, in prelude to human clinical trials for there ability to ameliorate HIV infection through the prevention of viral integration (Figure 2). Perhaps a combination of HAART and a VPAC2-specific agonist would have increased efficacy for the treatment of HIV infection. Ideally, activators of VPAC2 should be designed so as not to penetrate the blood-brain barrier to avoid central nervous system adverse effects as VPAC2 is widely expressed in the brain [7].

## 8. Conclusions

VPAC1 and VPAC2 receptors are able to regulate the integration of the HIV viral cDNA. Despite a lack of current research to further document the regulatory effects of VPAC1 and VPAC2 stimulation on HIV integration, development of specific inhibitors of VPAC1 signal transduction as well as further research to develop more specific activators of VPAC2 signaling, including drug discovery for small-molecular-weight, non-peptide inhibitors, is warranted. Future therapeutics that target VPAC1 and/or VPAC2 are predicted to have significant value for the treatment and possibly prevention of HIV/AIDS, something that is urgently needed.

## References

[1] Gottlieb M.S., Schroff R., Schanker H.M., Weisman J.D., Fan P.T., Wolf R.A., Saxon A. (1981). Pneumocystis carinii pneumonia and mucosal candidiasis in previously healthy homosexual men: evidence of a new acquired cellular immunodeficiency. N. Engl. J. Med..

[2] http://www.unaids.org/globalreport/Global_report.htm accessed on 9 March 2011

[3] Cock K.M., Weiss H.A. (2000). The global epidemiology of HIV/AIDS. Trop. Med. Int. Health.

[4] Temesgen Z. (1999). Overview of HIV infection. Ann. Allergy Asthma Immunol..

[5] Harmar A.J., Arimura A., Gozes I., Journot L., Laburthe M., Pisegna J.R., Rawlings S.R., Robberecht P., Said S.I., Sreedharan S.P. (1998). International union of pharmacology. XVIII. Nomenclature of receptors for vasoactive intestinal peptide and pituitary adenylate cyclase-activating polypeptide. Pharmacol. Rev..

[6] Ulrich C.D., Holtmann M., Miller L.J. (1998). Secretin and vasoactive intestinal peptide receptors: members of a unique family of G protein-coupled receptors. Gastroenterology.

[7] Dickson L., Finlayson K. (2009). VPAC and PAC receptors: From ligands to function. Pharmacol. Ther..

[8] Goetzl E.J., Pankhaniya R.R., Gaufo G.O., Mu Y., Xia M., Sreedharan S.P. (1998). Selectivity of effects of vasoactive intestinal peptide on macrophages and lymphocytes in compartmental immune responses. Ann. NY Acad. Sci..

[9] Lara-Marquez M., O'Dorisio M., O'Dorisio T., Shah M., Karacay B. (2001). Selective gene expression and activation-dependent regulation of vasoactive intestinal peptide receptor type 1 and type 2 in human T cells. J. Immunol..

[10] Bokaei P.B., Ma X.Z., Byczynski B., Keller J., Sakac D., Fahim S., Branch D.R. (2006). Identification and characterization of five-transmembrane isoforms of human vasoactive intestinal peptide and pituitary adenylate cyclase-activating polypeptide receptors. Genomics.

[11] Nicole P., Du K., Couvineau A., Laburthe M. (1998). Site-directed mutagenesis of human vasoactive intestinal peptide receptor subtypes VIP1 and VIP2: Evidence for difference in the structure-function relationship. J. Pharmacol. Exp. Ther..

[12] Xia M., Gaufo G.O., Wang Q., Sreedharan S.P., Goetzl E.J. (1996). Transduction of specific inhibition of HuT 78 human T cell chemotaxis by type I vasoactive intestinal peptide receptors. J. Immunol..

[13] Gourlet P., Vandermeers A., Vertongen P., Rathe J., De Neef P., Cnudde J., Waelbroeck M., Robberecht P. (1997). Development of high affinity selective VIP1 receptor agonists. Peptides.

[14] Robberecht P., Waelbroeck M., Dehaye J.P., Winand J., Vandermeers A., Vandermeers-Piret M.C., Christophe J. (1984). Evidence that helodermin, a newly extracted peptide from Gila monster venom, is a member of the secretin/VIP/PHI family of peptides with an original pattern of biological properties. FEBS Lett..

[15] Robberecht P., Gourlet P., Vertongen P., Svoboda M. (1996). Characterization of the VIP receptor from SUP T1 lymphoblastrs. Adv. Neuroimmunol..

[16] Gourlet P., Vertongen P., Vandermeedrs A., Vandermeers-Piret M.C., Rathe J., De Neef P., Waelbroeck M., Robberecht P. (1997). The long-acting vasoactive intestinal polypeptide agonist RO 25-1553 is highly selective for the VIP2 receptor subclass. Peptides.

[17] Murphy T.J., Alexander R.W., Griendling K.K., Runge M.S., Bernstein K.E. (1991). Isolation of a cDNA encoding the vascular type-1 angiotensin II receptor. Nature.

[18] Mukoyama M., Nakajima M., Horiuchi M., Sasamura H., Pratt R.E., Dzau V.J. (1993). Expression cloning of type 2 angiotensin II receptor reveals a unique class of seven-transmembrane receptors. J. Biol. Chem..

[19] Pert C.B., Ruff M.R., Hill J.M. (1988). AIDS as a neuropeptide disorder: peptide T, VIP, and the HIV receptor. Psychopharmacol. Bull..

[20] Gilles A., Miquiles A., Luis J., Faure E. (1998). Activation of transcription from the human Immunodeficiency virus type1 (HIV-1) long terminal repeat by the vasoactive intestinal peptide (VIP). Ital. J. Biochem..

[21] Veljkovic V., Metlas R., Raspopovic J., Pongor S. (1992). Spectral and sequence similarity between vasoactive intestinal peptide and the second conserved region of human immunodeficiency virus type 1 envelope glycoprotein (gp120): possible consequences on prevention and therapy of AIDS. Biochem. Biophys. Res. Commun..

[22] Djordjevic A., Veljkovic M., Antoni S., Sakarellos-Daitsiotis M., Krikorian D., Zevgiti S., Dietrich U., Veljkovic N., Branch D.R. (2007). The presence of antibodies recognizing a peptide derived from the second conserved region of HIV-1 gp120 correlates with non-progressive HIV infection. Curr. HIV Res..

[23] Branch D.R., Valenta L.J.E., Yousefi S., Sakac D., Singla R., Bali M., Sahai B.M., Ma X.Z. (2002). VPAC1 is a cellular neuroendocrine receptor expressed on T cells that actively facilitates productive HIV-1 infection. AIDS.

[24] Koh S.W. (1991). Signal transduction through the vasoactive intestinal peptide receptor stimulates phosphorylation of the tyrosine kinase pp60c-src. Biochem. Biophys. Res. Commun..

[25] Branch D.R. (2011). Role of G protein-coupled vasoactive intestinal peptide receptors in HIV integration. Fut. HIV Ther..

[26] Gallay P., Swingler S., Song J., Bushman F., Trono D. (1995). HIV nuclear import is governed by the phosphotyrosine-mediated binding of matrix to the core domain of integrase. Cell.

[27] Camaur D., Gallay P., Swingler S., Trono D. (1997). Human immunodeficiency virus matrix tyrosine phosphorylation: characterization of the kinase and its substrate requirements. J. Virol..

[28] Tsutsumi M., Claus T.H., Liang Li Y, Yang L., Zhu J., Dela Cruz F., Peng X., Chen H., Yung S.L., Hamren S. (2002). A potent and highly selective VPAC2 agonist enhances glucose-induced insulin release and glucose disposal: A potential therapy for type 2 diabetes. Diabetes.

[29] Yung S.L., Dela Cruz F., Hamren S., Zhu J., Tsutsumi M., Bloom J.W., Caudle M., Roczniak S., Todd T., Lemoine L. (2003). Generation of highly selective VPAC2 receptor agonists by high throughput mutagenesis of vasoactive intestinal peptide and pituitary adenylate cyclase-activating peptide. J. Biol. Chem..

[30] Bokaei P.B., Ma X.Z., Sakac D., Branch D.R. (2007). HIV-1 integration is inhibited by stimulation of the VPAC2 neuroendocrine receptor. Virology.

[31] Pohl M., Wank S.A. (1998). Molecular cloning of the helodermin and exendin-4 cDNAs in the lizard. Relationship to vasoactive intestinal polypeptide/pituitary adenylate cyclase activating polypeptide and glucagons-like peptide 1 and evidence against the existence of mammalian homologues. J. Biol. Chem..

[32] Langer I., Gregoire F., Nachtergael I., De Neef P., Vertongen P., Robberecht P. (2004). Hexanoylation of a VPAC2 receptor-preferring ligand markedly increased its selectivity and potency. Peptides.

[33] Pan C.Q., Li F., Wang W., Dumas M., Froland W., Yung S.L., Li Y., Roczniak S., Claus T.H., Wang Y.J., Whelan J.P. (2007). Engineering novel VPAC2-selective agonists with improved stability and glucose-lowering activity *in vivo.*. J. Pharmacol. Exp. Ther..

[34] Tannu S.A., Renzetti L.M., Tare N., Ventre J.D., Lavelle D., Lin T.A., Morschauser A., Paciorek J., Bolin D.R., Miche H. (2010). Dual bronchodilatory and pulmonary anti-inflammatory activity of RO5024118, a novel agonist at vasoactive intestinal peptide VPAC receptors. Br. J. Pharmacol..

[35] Ma Y., Ma M., Dai Y., Hong A. (2010). Expression, identification and biological effects of a novel VPAC2-specific agonist with high stability and bioactivity. Acta Biochim. Biophys. Sin. (Shanghai).

